# Identifying inhibitors of β-haematin formation with activity against chloroquine-resistant *Plasmodium falciparum* malaria parasites via virtual screening approaches

**DOI:** 10.1038/s41598-023-29273-w

**Published:** 2023-02-14

**Authors:** Leah Amod, Roxanne Mohunlal, Nicole Teixeira, Timothy J. Egan, Kathryn J. Wicht

**Affiliations:** 1 Department of Chemistry, University of Cape Town, Rondebosch, 7701 South Africa; 2 Drug Discovery and Development Centre (H3D), University of Cape Town, Rondebosch, 7701 South Africa; 3 Institute of Infectious Diseases and Molecular Medicine, University of Cape Town, Rondebosch, 7701 South Africa

**Keywords:** Cell biology, Chemical biology, Computational biology and bioinformatics, Drug discovery, Structural biology, Inorganic chemistry, Chemistry, Medicinal chemistry, Chemical libraries, Cheminformatics, Drug discovery and development, Screening, Target validation

## Abstract

The biomineral haemozoin, or its synthetic analogue β-haematin (βH), has been the focus of several target-based screens for activity against *Plasmodium falciparum* parasites. Together with the known βH crystal structure, the availability of this screening data makes the target amenable to both structure-based and ligand-based virtual screening. In this study, molecular docking and machine learning techniques, including Bayesian and support vector machine classifiers, were used in sequence to screen the in silico ChemDiv 300k Representative Compounds library for inhibitors of βH with retained activity against *P. falciparum*. We commercially obtained and tested a prioritised set of inhibitors and identified the coumarin and iminodipyridinopyrimidine chemotypes as potent in vitro inhibitors of βH and whole cell parasite growth.

## Introduction

Malaria is an ancient parasitic infection caused by five virulent species of the genus *Plasmodium*, the most lethal of which is *Plasmodium falciparum* (*Pf*)^1^. Over the past several decades, the emergence of antimalarial-resistant strains of *Pf* has countered unified efforts to control the disease, and recent reports of partial artemisinin resistance in East Africa have spurred much concern^2,3^. Although antimalarial research has produced numerous lead candidates, conventional drug discovery strategies such as high-throughput screening (HTS) are notoriously expensive and high risk, and novel drugs have been slow to enter the clinical market^4^. In silico screening is now a widely used tactic for fast-tracking drug development. With the use of either structure-based or ligand-based methods, large chemical libraries can be virtually screened for compounds most likely to show activity, resulting in significant enrichment rates relative to random screening^5^.

It is now well-accepted that the 4-aminoquinoline antimalarials, some of which have been rendered ineffective by resistance in certain regions, act by inhibiting haemozoin (HZ) formation^6^. During the asexual blood stage of the *Plasmodium* life cycle, haemoglobin is catabolised within the parasite’s digestive vacuole, releasing free haem as a by-product. Haem has the tendency to partition into cell membranes and catalyses the formation of reactive oxygen species; hence, *Plasmodium* has evolved a complex mechanism of haem detoxification that incorporates the conversion of haem to inert HZ crystals. Given that the combined potency and low cost of chloroquine (CQ), one of the most successful drugs in this class, has yet to be emulated by other clinical antimalarials^7^, there has been considerable interest in discovering alternative HZ-inhibiting chemotypes. More importantly, compared to other validated targets, HZ has the distinct advantage of not being a gene product, and is thus immutable. Resistance to HZ inhibitors is conferred by mutations in membrane transport proteins that cause an efflux of the drug away from the digestive vacuole, and the structure-specific nature of this mechanism reduces the likelihood of cross-resistance with chemically unrelated HZ inhibitors^8^. Consequently, a detergent-mediated, biomimetic assay for quantifying β-haematin (synthetic HZ, βH) inhibition was developed and adapted for HTS^9–11^. These screens have been successful in identifying potent βH-inhibiting chemotypes that are active on whole cell *Pf* cultures, including the benzimidazoles, benzamides and triaryl imidazoles^12–15^.

The crystal structure and morphology of βH has also been pertinent in elucidating the mode of action of the 4-aminoquinolines and other βH-inhibiting compounds^16^. βH comprises centrosymmetrically related haematin ([Fe(III)PPIX]) units which dimerise via two reciprocal iron–carboxylate bonds. In turn, the dimers stack via hydrogen bonds to form parallel strands of porphyrin units^17^. Buller et al. proposed a non-covalent binding site on the (001) face, which is not only the fastest-growing face and thus the most efficient site for inhibition, but the corrugated surface also exposes chemical groups and aromatic surfaces that favour the adsorption of inhibitors^16^.

The knowledge of the βH crystal structure and the availability of HTS βH inhibition data makes the target amenable to both structure- and ligand-based virtual screening (S- and LBVS), and in fact, both strategies have previously achieved very promising results. Molecular docking is a structure-based approach that is most commonly used to model the interactions between a small molecule and a protein; however, it can be extended to other classes of therapeutic targets where the structure is known^18^. A set of drug-like compounds from the ZINC15 database was docked against the βH crystal structure and a small selection of high-ranking compounds was prioritized for experimental testing, resulting in a hit rate of 20% for βH inhibition ≤150 µM^19^. However, in vitro βH inhibitors are not guaranteed to show activity against whole-cell *Pf*, and the mechanisms underpinning HZ inhibition are not yet sufficiently understood to rationalise by inspection whether or not a βH inhibitor will be active against the parasite. Furthermore, there are multiple factors that affect the ability of the compound to access and accumulate at the site of the haem target in the DV, so it is uncommon to see direct correlations between βH inhibition activity and whole-cell activity^20,21^. In this regard, LBVS is an attractive strategy for enriching antiplasmodium hit rates. Typically used in the absence of a 3D target structure, ligand-based methods analyse known small-molecular inhibitors and attempt to correlate their specific structural and physiochemical features with a desired biological activity. Quantitative structure-activity relationships, pharmacophore modelling, and machine learning classifiers are types of ligand-based methods that are commonly used in drug design^22^. Previously, Bayesian models were built to predict βH-inhibition and antiplasmodium activity and achieved hit rates of 25% and 33%, respectively^23^.

In this study, we employ a two-step virtual screening workflow involving molecular docking and machine learning methods to screen a commercial library for βH inhibitors with retained activity against cultures of *Pf*.

## Results and discussion

### Molecular docking

**Figure 1:**
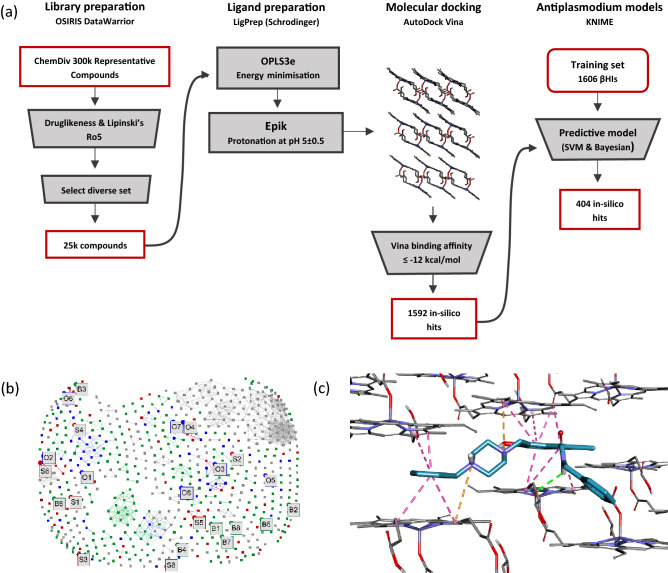
Virtual screening of the ChemDiv 300k Representative Compounds library and prioritisation of in-silico hits for purchasing and experimental testing against βH and whole-cell cultures of *Pf*. (**a**) Summarised computational workflow for predicting βH inhibition and antiplasmodium activity. (**b**) Similarity map of the 374 active compounds from the training set (black) and the 404 LBVS hits, which are categorised as SVM (**S**, in red), Bayesian (**B**, in green), and overlap (**O**, in blue) based on which model(s) predicted them active, overlayed in chemical space. The map assisted in prioritising a set of 24 structurally unrelated compounds which excluded those scaffolds that have been previously investigated as βH inhibitors, i.e., those that appear frequently in the training set actives. (**c**) Purchased compound **S4** docked at (001) face of βH. **S4** forms multiple π-π stacking interactions (pink dashed line) with the parallel porphyrin rings and an (amine)NH···O(carboxyl) hydrogen bond (green dashed line).

Molecular docking was used to predict the interaction strength of 25,000 compounds with the in silico βH crystal structure (Fig. 1A). The ChemDiv 300k Representative Compounds library was initially filtered for druglikeness using Lipinski’s Rule of Five and OSIRIS *DataWarrior*’s^24^ inhouse score. To further reduce the computational expense of the virtual screen, the “select diverse set” feature in *DataWarrior*, which uses a fragment-based molecular descriptor to compute structural similarity, was used to obtain a set of 25,000 prioritised compounds. Using Schrodinger’s LigPrep^25^, the ligands were prepared for docking by performing an energy minimisation and generating their protonated states at pH 5, in accordance with the acidity of the parasite’s digestive vacuole. The ligands were then docked using AutoDock Vina^26^. A cut-off of − 12 kcal mol^−1^ was chosen by fitting the docking scores to a cubic function and rounding the inflection point of −12.9 kcal mol^−1^ up to the nearest whole number (Supplementary Fig. S1A), which classified 1592 molecules (6.4%) as “docking hits” or predicted βH inhibitors. The docking poses show that compounds binding at the (001) face, or the opposite (00$\stackrel{\mathrm{-}}{1}$) face have stronger binding affinities, which can be explained by the parallel porphyrin rings and free carboxyl groups that are available for forming π–π stacking interactions and hydrogen bonds, respectively (Supplementary Fig. S1B). Furthermore, the 25,000 compounds were mapped in chemical space using principal component analysis (PCA, Supplementary Fig. S1C), which showed the high-ranking compounds to be negatively shifted in PC1 relative to those with poorer, less negative scores. Based on the PC loadings, this corresponds to molecules with higher logP values and more aromatic rings (Supplementary Table S1), which supports the importance of π–π stacking.

### Building models and in-silico screening for antiplasmodium activity

Two classification models were trained with data from previous high-throughput screens for βH inhibitors (Supplementary Table S2) and used to predict which of the docking hits would be active against whole-cell cultures of *Pf*. The support vector machine (SVM) model was built using LibSVM, as implemented in the KNIME Analytics Platform^27,28^. The class-distinguishing ability of four kernel functions (linear, polynomial, radial basis function, sigmoidal) was evaluated by assembling a fivefold cross-validation loop; the training set was partitioned into five groups and for each iteration, one group was held out as the test set such that the activity of each molecule was predicted only once and a receiver operating characteristic (ROC) score for the model was obtained. The linear kernel function showed the best performance, which is not unexpected when modelling a high number of input features *N*. Here, these features included a total of 1875 1D, 2D, and 3D molecular descriptors and the extended connectivity fingerprint (ECFP6). The regularization parameter *C*, which guards against over-fitting by controlling the model’s tolerance for misclassified data, was optimised to a value of 0.274, which resulted in a ROC score of 0.935 (Supplementary Table S3). For the Bayesian Fingerprint model, the performance of various molecular fingerprints was evaluated, with the circular functional connectivity (FCFP6) descriptor achieving the best ROC score of 0.918 (Supplementary Table S4). Due to the reduced computational expense of building Bayesian models relative to the SVM, here the model was optimised via a leave-one-out cross-validation loop in which molecules were individually held out as the test set.

The optimised SVM and Bayesian models were then used screen the 1592 docking hits for antiplasmodium activity, which predicted a total of 404 of these (25%) to be bioactive. These were categorised as: SVM and Bayesian overlap (**O**), Bayesian only (**B**), or SVM only (**S**) based on which models predicted them to be active. Interestingly, when the models were used to predict the activity of the control compound, chloroquine (Supplementary Table S5), the SVM model falsely classified the drug as inactive. This could be explained by chloroquine’s long, flexible side chain at position 4 of the quinoline ring, which distinguishes it from the highly lipophilic, planar βH inhibitors that dominate the active class in the training set. In contrast, the Bayesian Fingerprint model correctly predicted chloroquine to be active.

Once the models had been optimised, the predictions made for the training set were analysed to identify substructures that appear frequently in each of the respective classes (Table 1). The Bayesian Fingerprint and SVM models detect the same fragments as being favourable for antiplasmodium activity, several of which comprise an *N*-heteroaromatic ring. The urea moiety is also significantly enriched in the active class. Conversely, the 1,4-dihydroquinoline scaffold and several of its derivatives frequently appear in the inactive class, as do the aryl sulfonamides.

**Table 1:** Fragments which are frequently predicted active (“good”) or inactive (“bad”) against *Pf* by the Bayesian and SVM classification models.

“Good” fragment	# Predicted active		# True active	“Bad” fragment	# Predicted active		# True active
	Bayesian	SVM			Bayesian	SVM	
	70/70	70/70	70/70		0/147	0/147	0/147
	56/56	56/56	56/56		6/146	1/146	6/146
	47/47	47/47	47/47		1/126	0/126	3/126
	56/57	53/57	53/57		1/117	0/117	3/117

**Figure 2:**
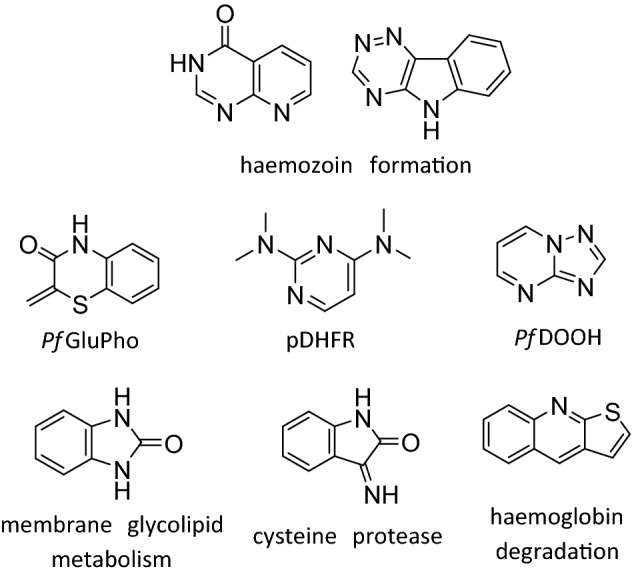
Scaffolds predicted bioactive by the SVM and/or Bayesian classification models that are not present in the training set but have documented activity against *Plasmodium* spp. Their proposed targets are given below.

The 404 in-silico hits comprise a total of 73 scaffolds, 41 of which are not present in the training set. However, 13 of these unique scaffolds have previously documented antiplasmodium activity (Fig. 2 and Supplementary Fig. 2), including the pyrido[1,3-*d*]pyrimidin-4-one and 1,2,4-triazino[5,6-*b*]indole scaffolds that were previously identified as haemozoin inhibitors. Other targets which have been proposed for particular scaffolds (Fig. 2) include deoxyhypusine hydroxylase, glucose-6-phosphate dehydrogenase, dihydrofolate reductase, cysteine protease, and other elements of the haem detoxification pathway, while the modes of the action for the remaining scaffolds have not yet been elucidated (Supplementary Fig. 2)^29–35^.

### Similarity analysis, visual inspection of docking poses, and compound selection

**Figure 3:**
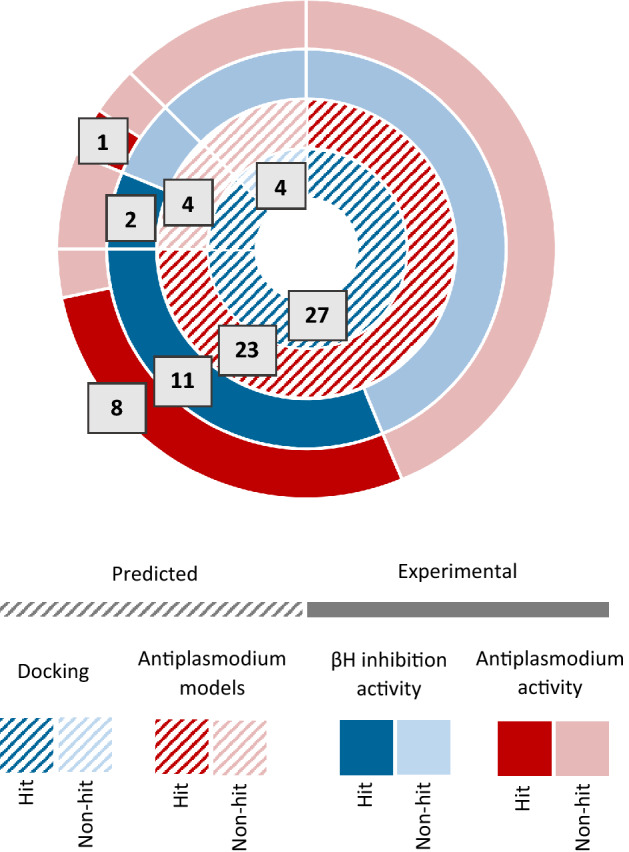
Graphic representation of the computational and experimental results for 31 compounds purchased from ChemDiv.

All 404 compounds could not be purchased, so further criteria were introduced to prioritise compounds for experimental testing. Firstly, the compounds were ranked by their SVM and Bayesian scores and the top compounds were examined. This set had limited structural diversity and was notably dominated by chemotypes with well-documented βH inhibition and antiplasmodium activity, i.e., quinolines and benzimidazoles. Thus, a similarity map of both the LBVS hits and the actives –from the training set was generated in *OSIRIS* DataWarrior^24^ (Fig. 1B) to assist in the selection of a structurally diverse, representative set of compounds that was enriched in novel chemotypes. Compounds of the same chemical class were distinguished by their in-silico scores, as well as their docking poses, which were inspected in Discovery Studio Visualizer^36^. Preference was given to compounds showing multiple π–π stacking interactions with the porphyrin rings of the crystal and/or hydrogen bonds with the free carboxyl side chains, as well as those docking to the crystal via two haematin units, which is facilitated by a twist between the two aromatic moieties in the molecule (Fig. 1C). A total of 31 compounds were purchased, which included eight compounds from each of the **O** and **B** categories, seven from the **S**, and eight classified as “non-hits”. Four of these were docking hits that were predicted inactive by both antiplasmodium models, and the remaining docked with poor scores between –4.5 and –6 kcal mol^−1^ to the in silico βH crystal structure (Fig. 3 and Supplementary Table S5).

### NP-40 detergent-mediated assay for βH inhibition

The compounds were tested for activity against βH at a concentration range of 0–500 µM, using the detergent-mediated and colorimetric pyridine ferrochrome methods described by Carter et al. and Ncokazi and Egan, respectively (Supplementary Table S6)^9,10^. As expected, the four compounds that received poor docking scores (−4.5 to −6 kcal mol^−1^) showed no activity against βH up to a concentration of 500 µM. Of the 27 compounds classified as docking hits, 11 showed IC_50_ values < 150 µM, corresponding to a hit rate of 41% for βH inhibition. Five compounds showed IC_50_ values comparable to CQ (16-40 μM) and **O3** and **I6** showed superior activity with IC_50_s of 8 and 11 μM, respectively. Nine of the eleven βH inhibitors were classified as LBVS hits (Table 2).

**Table 2:** Experimentally active βH inhibitors purchased from ChemDiv, prioritised by molecular docking and machine learning models.

Compound code	Structure	NP-40 βH IC_50_ (µM)	*Pf* NF54 IC_50_ (µM)	*Pf* Dd2 IC_50_ (µM)	RI
**O3**		8	0.154	0.244	1.6
**O5**		129	–	–	–
**O8**		129	3.545	3.320	0.9
**B1**		37	1.610	0.971	0.6
**B2**		24	2.171	3.010	1.4
**S1**		30	0.158	0.545	3.4
**S3**		40	Inactive	–	–
**S4**		82	2.08	1.78	0.9
**S5**		16	6.14	5.80	0.9
Chloroquine		25	0.014	0.376	26.7

### pLDH assay for *Pf* growth inhibition

All 31 purchased compounds were tested at concentrations of 1 and 5 µM against the CQ-sensitive *Pf* NF45 strain, using the parasite lactate dehydrogenase (pLDH) assay described by Makler et al (Supplementary Table S7 and S8)^35,37^. Interestingly, one compound predicted to be inactive against parasites by both the SVM and Bayesian models showed moderate growth inhibition (∼ 60%) at both tested concentrations. This compound was not a βH inhibitor, so it likely acts against other biological targets or via a different pathway. Since the antiplasmodium models were trained only on βH inhibiting active compounds, this would explain why neither model predicted this compound to be active. Of the nine βH inhibitors, eight inhibited *Pf* growth >50% at 5 µM. Excluding **O5**, the activity of these compounds was fully ascertained by performing dose-response assays against the *Pf* NF54 and CQ-resistant *Pf* Dd2 strains (Supplementary Table S8). Six compounds showed IC_50_ values ≤ 5 against both strains, corresponding to an overall hit rate of 30% for antiplasmodium activity. Furthermore, compound **B1** showed sub-micromolar activity against the Dd2 strain and compounds **O3** and **S1** showed sub-micromolar activity against both the NF54 and Dd2 strains (Table 2). The primary mode of action for these compounds is presumed to be via the inhibition of hemozoin formation and a subsequent increase in cytotoxic haem or drug-haem complexes, however the disruption of other biological pathways may contribute to their activity. This would need further investigation on a case-by-case basis to validate the biological mechanisms or involvement of protein targets for each compound.

Interestingly, compounds **O3** and **O8** both contain a coumarin ring system. The structural diversity and abundance of coumarins isolated from natural sources has drawn particular interest from the pharmaceutical industry. The coumarin core is considered a privileged scaffold, particularly since these compounds have displayed antimicrobial, anticancer, and antioxidant activities. In addition to some coumarin-containing plant extracts, synthetic coumarin derivatives have been investigated as potential antimalarial agents, with success mostly being found via molecular hybridization. For example, chalcone-triazole- and ferrocenyl-oxazine-coumarins were found to inhibit *Pf* growth in the low micromolar range. The respective authors cite falcipain-2 inhibition and DNA binding as potential targets, with haemozoin inhibition also implicated for the latter series^38,39^. The training set contains a total of fifteen coumarins, which all showed βH IC_50_ values < 60 μM, but are inactive against whole-cell Pf cultures. It is interesting that, despite the absence of this chemotype in the “active” training set, both antiplasmodium models identified two experimentally active coumarin-containing molecules. Further, of the 81 coumarins classified as docking hits, an additional 17 were predicted active by the SVM and/or Bayesian models.

Compound **S1** contains the iminodipyridinopyrimidine (IDPP) core and is particularly interesting in that there has been very little investigation into the antimalarial potential of this scaffold. However, **S1** is reported as antimalarially active in the PubChem BioAssay database and was found to inhibit parasite growth in the first generation (48 h incubation) with an EC_50_ of 0.5 µM (AID 504832). Several analogues of these compounds are also contained in the BioAssay database and have exhibited good to moderate activities against *Pf* 3D7 and Dd2 (AID 2302 & 2306), although the biological target and structure-activity relationships of this compound class have yet to be investigated. The IDPP scaffold was not present in the training set. Twenty-nine IDPPs were present in the set of 25 000 compounds screened, with seven having docking scores ≤ –12 kcal/mol. Of these, only **S1** and its analogue were predicted bioactive by either classification model. These differ from the remaining five IDPPs in the presence of a basic nitrogen on the substituent of the dihydropyridyl ring, which is predicted to be ~ 95% protonated at pH 5, and only 15–18% protonated at pH 7. Chloroquine’s potency is often partially attributed to ‘pH trapping’, the mechanism by which the drug accumulates in the parasite’s digestive vacuole. It is possible that the SVM model implicitly characterised a molecule’s propensity for pH trapping as being important for antiplasmodium activity.

## Conclusions

Modern drug discovery is becoming increasingly in silico based to mitigate the exorbitant costs of conventional HTS. This study capitalises on the previously solved βH crystal structure and the abundance of publicly available HTS data by combining SB- and LBVS techniques to identify βH inhibitors with retained activity against *Pf*. This two-step workflow achieved excellent enrichment rates for βH inhibition relative to random screening. The antiplasmodium SVM model in particular was successful in identifying three compounds with sub-micromolar activity against *Pf*. The coumarin and IDPP scaffolds represent promising starting points for lead optimisation and merit further pharmacological investigation.

## Computational and experimental methods

### Molecular docking for potential β-haematin inhibitors

#### Preparing the commercial library

Hierarchical virtual screening was carried out on the ChemDiv 300k Representative Compounds Library^40^; first by filtering for druglikeness in OSIRIS DataWarrior^24^. Lipinski’s Rule of Five, a widely used estimator for oral bioavailability, was applied to exclude molecules in violation of any the following criteria: molecular weight ≤ 500 Da, number of hydrogen bond donors ≤ 5, number of hydrogen bond acceptors ≤ 10, octanol-water partition coefficient (logP) ≤ 5. DataWarrior’s in-house ‘druglikeness’ score was used as an additional filter, excluding molecules that received < 0. Finally, the ‘select diverse set’ feature was implemented to obtain a small representative library of 25,000 molecules.

#### Preparing ligands for molecular docking

The filtered library was prepared for molecular docking in Maestro, an interface for the Schrödinger computational platform^41^. LigPrep was used to convert the 2D structures to their energy minimised 3D conformers with the OPLS3e force field^25^. Using Epik, the protonated species were generated at pH 5.0 ± 0.5, based on the pH of the biological target of interest i.e., the *Pf* digestive vacuole^42^.

#### The receptor model for β-haematin

Using a modified cvff force field, the βH µ-propianato dimer was optimised by a group of researchers at Stellenbosch University with the BIOVIA Materials Studio package^43,44^. A receptor model expressing the dominant (100), (010) and fastest-growing (001) faces was generated, using the same software, by growing a 3 × 3 × 3 ‘supercell’ and exporting it as a Protein Data Bank (pdb) file.

#### Molecular docking against the β-haematin crystal structure

Docking was performed in the Python Prescription Virtual Screening Tool (PyRx), which compiles several open-source programs into one user-friendly interface, including OpenBabel and AutoDock Vina^26^. As required by AutoDock Vina, all receptor and ligand structures were converted to pdbqt format, an extension of the pdb format with partial charges (Q) and atom types (T) defined. Within the Vina wizard, the search space was set to enclose the entirety of the crystal surface; centre (x, y, z): (13.5, 22.5, 12) and dimensions (x, y, z): (48, 47.5, 43). Each ligand was docked at an exhaustiveness of 8 and only the lowest energy binding mode was retained. Upon visual inspection of the docking scores, a Vina binding affinity of −12 kcal mol^−1^ was chosen as the cut-off for hit selection, classifying 1592 compounds as potential βH inhibitors.

### Classification models for predicting antiplasmodium activity

The antiplasmodium models were built in the KNIME Analytics Platform v4.3.3^27^. Though not developed specifically for drug discovery, KNIME’s collaborative philosophy has meant that several cheminformatics platforms, including RDKit and the Chemistry Development Kit (CDK), have integrations within the platform. Together with its drag-and-drop style graphical interface, this makes KNIME an attractive machine learning tool for non-experts.

#### Training data

The training data was largely sourced from a HTS for βH-inhibiting antimalarials, piloted by Vanderbilt University (VU)^15^. Only βH inhibitors (βH IC_50_ ≤ 100 µM) which had been tested against *Pf* were included; molecules were considered ‘active’ (1) if they exhibited *Pf* IC_50_ ≤ 1 µM and ‘inactive’ (0) if *Pf* IC_50_ ≥ 1.5 µM. The same criteria were applied to ∼100 bioactive neo- and isocryptolepine derivatives synthesised by a group at Okayama University (OU)^45,46^. In addition, a number of molecules from the Tres Cantos Antimalarial Compounds Set (TCAMS)^47^ were included in the training set using unpublished βH inhibition data; these molecules all exhibited ≥ 90% βH inhibition and were considered active if they inhibited ≥ 90% *Pf* growth, both relative to the chloroquine control drug. The resultant set contained a total of 1606 molecules, with 374 actives (Supplementary Table S2). For the SVM model, the training molecules were converted to their 3D energy minimised representations at pH 5 ± 0.5 in LigPrep and multiple protonation states were retained, resulting in 1707 data instances.

#### Molecular descriptors

Five types of molecular fingerprints were generated with the CDK’s Fingerprints node in KNIME. In addition, a total of 1875 molecular descriptors (1D, 2D and 3D) were calculated using the open-source software PaDEL.

#### Support vector machine (SVM)

The C-SVM classifier was built using LibSVM, an open-source library that has its learning code implemented in KNIME^28^. The input features were prepared by (a) expanding the ECFP6 fingerprint into a series of 1024 integers and (b) normalising the 1875 descriptors calculated in PaDEL. Since there is no sure method for predicting which kernel function will perform best for a given dataset, each kernel implemented in LibSVM (linear, polynomial, RBF, sigmoidal) was evaluated via a fivefold cross-validation loop with stratified sampling. The relevant hyperparameters were optimised for each kernel via the hillclimbing method by incorporating a parameter optimisation loop.

#### Bayesian classification model

The Bayesian classifier was built using the Fingerprint Bayesian Learner and Predictor nodes. Generally, Bayesian models utilise a naïve, Laplacian-corrected algorithm based on Bayes theorem of conditional probability.

1 $$P\left(A|B\right)= \frac{P(B|A)\cdot P(A)}{P(B)}$$

where P(A|B) is the probability of a compound being active given the presence of a molecular feature, P(B|A) is the likelihood of a feature being present in an active compound, P(A) is the probability of a compound in the training set being active, and P(B) is the probability of a feature being present in the training set. The classifier ‘naively’ assumes that the input features are independent and multiplies the probabilities of the individual events. However, the frequency of features in the training set is accounted for by introducing a Laplacian-corrected estimator, so that the score is given as a sum of the corrected estimators. Models were built for five molecular fingerprints implemented in the CDK and evaluated by a leave-one-out cross validation loop in which molecules are individually held out and classified using the remaining data instances.

### NP-40 detergent mediated assay for βH formation

A set of 31 compounds was purchased based on their in-silico scores, structural diversity, and availability. The βH inhibition activity of the purchased compounds was investigated using the detergent-mediated NP-40 assay developed by Carter et al. in 96-well plates^10^. The assay was analysed using the pyridine-ferrochrome method described by Ncokazi and Egan^9^. The UV-vis absorbance was read at 405 nm on a Thermo Scientific Multiskan GO plate reader and the IC_50_ values were calculated by plotting sigmoidal dose-response curves in GraphPad Prism v 9.0.0. (GraphPad Software Inc., La Jolla, CA, USA).

### Parasite lactate dehydrogenase assay for antiplasmodium activity

The growth inhibition activity of the compounds was tested against two *Pf* strains: CQ-sensitive NF54 and CQ-resistant Dd2 cell lines. Dose response activity was measured with the pLDH assay in 96-well plates, as described by Makler et al. in 96-well plates^37^. The UV-vis absorbance was read at 620 nm on a MultiSkan Go plate reader and IC_50_ values were determined using non-linear dose-response analysis in GraphPad Prism v 9.0.0.

## Supplementary Information

Supplementary Information 1.

Supplementary Information 2.

## Data Availability

The datasets used and/or analysed during the current study are available in the supplementary information files or from the corresponding author on reasonable request.
