# Supplementary material for: Identifying inhibitors of β-haematin formation with activity against chloroquine-resistant Plasmodium falciparum malaria parasites via virtual screening approaches
Source: Sci Rep. 2023 Feb 14;13:2648. doi: 10.1038/s41598-023-29273-w (PMC9929333; doi:10.1038/s41598-023-29273-w)
Supplement: Supplementary file 2 — Supplementary Information 2. [file 41598_2023_29273_MOESM2_ESM.docx]

**Supplementary information**

to

**Identifying inhibitors of β-haematin formation with activity against chloroquine-resistant *Plasmodium falciparum* malaria parasites via virtual screening approaches**

**Leah Amod^1^, Roxanne Mohunlal^1^, Nicole Teixeira^1^, Timothy J. Egan^1,3^, Kathryn J. Wicht^1,2,3^***

^1^Department of Chemistry, University of Cape Town, Rondebosch, 7701, South Africa

^2^ Drug Discovery and Development Centre (H3D), University of Cape Town, Rondebosch, 7701, South Africa

^3^ Institute of Infectious Diseases and Molecular Medicine, University of Cape Town, Rondebosch, 7701, South Africa

*kathryn.wicht@uct.ac.za

**Figure S1**. (a) Vina binding affinities for 25 000 compounds docked against the βH crystal structure and choice of cut-off for “docking hit” classification. (b) Top-ranked compounds docked to the (001) and (00$\bar{\text{1}}$) faces of the βH crystal surface. (c) PCA map showing the 25 000 compounds that were docked against the βH crystal structure. The highest-ranking compounds cluster in a distinct region of chemical space.

Vina binding affinity (kcal/mol)

Compound number


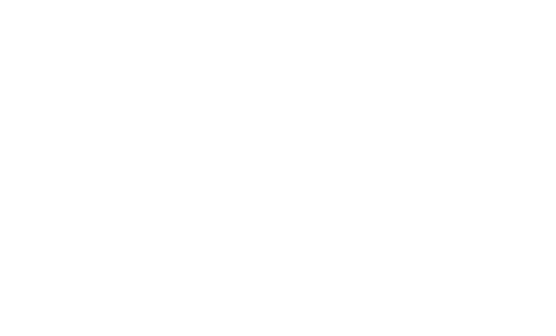


(a)

(b)


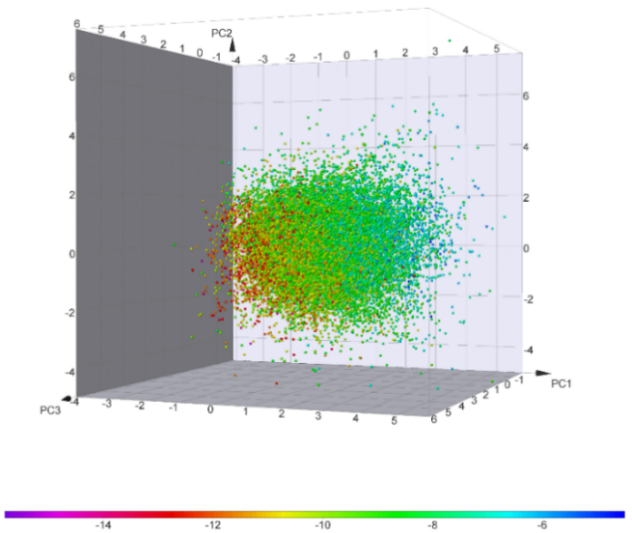

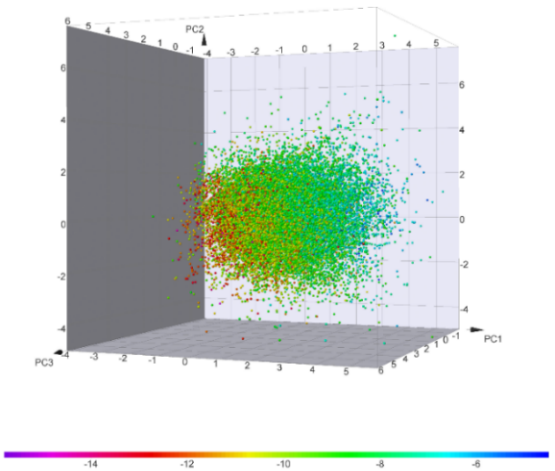


Vina binding affinity (kcal/mol)

(c)


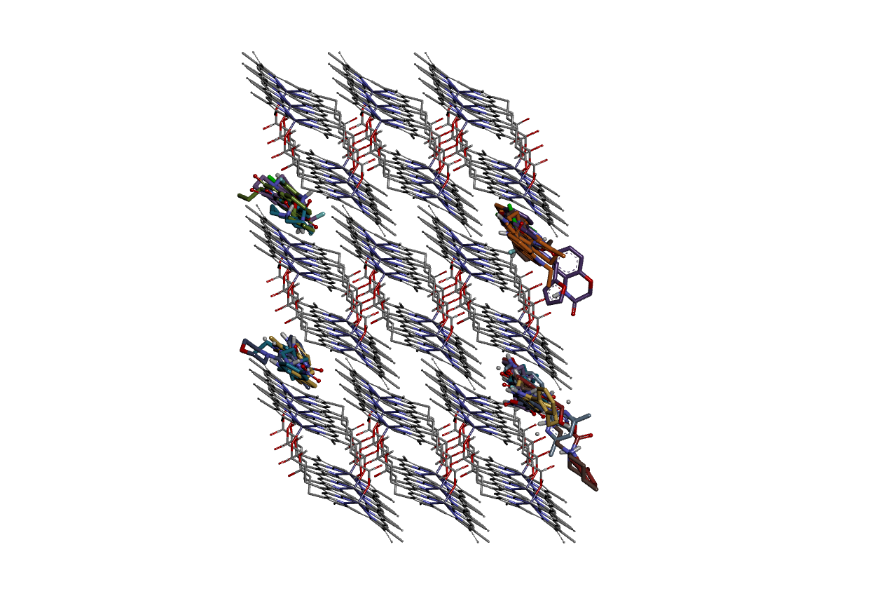


PC2

PC1

PC3

-14 -12 -10 -8 -6

**Table S1.** PCA loadings for the molecular descriptors used to map the virtual library in chemical space.

|  | **PC1** | **PC2** | **PC3** |
| --- | --- | --- | --- |
| **cLogP** | -0.590 | -0.140 | -0.142 |
| **#Aromatic rings** | -0.410 | -0.433 | 0.154 |
| **Molecular weight** | 0.009 | -0.346 | 0.278 |
| **#Basic nitrogens** | 0.064 | -0.178 | 0.524 |
| **#H-Donors** | 0.243 | -0.089 | 0.645 |
| **#Stereo centres** | 0.314 | 0.449 | 0.037 |
| **Relative PSA** | 0.328 | -0.477 | -0.364 |
| **#H-Acceptors** | 0.461 | -0.452 | -0.230 |

**Table S2.** Training data for predicting the antiplasmodium activities of βH inhibitors.

| Library | # Actives (1) | # Inactives (0) | Total |
| --- | --- | --- | --- |
| VU | 51 | 1224 | 1273 |
| OU | 94 | 8 | 102 |
| TCAMS | 229 | 0 | 229 |
| Total | 374 | 1232 | 1606 |

| **Kernel function** | **C** | **Gamma** | **Coef0** | **ROC score** |
| --- | --- | --- | --- | --- |
| **Linear** | **0.274** | NA | NA | **0.935** |
| Polynomial | 0.502 | 0.089 | 0.093 | 0.933 |
| RBF | 0.639 | 0.049 | NA | 0.931 |
| Sigmoidal | 0.368 | 0.075 | 0.097 | 0.461 |

**Table S3.** Optimisation of the C-SVM classifier for antiplasmodium activity. The linear kernel function (bold) showed the best receiver operating characteristic (ROC) score.

**Table S4.** Optimisation of the Bayesian Fingerprint Learner for antiplasmodium activity. The circular FCFP6 (bold) gave the best ROC score.

| Type | Fingerprint | ROC score |
| --- | --- | --- |
| Structural keys | MACCS | 0.849 |
|  | PubChem | 0.864 |
| Path-based | Standard | 0.870 |
| **Circular** | ECFP6 | 0.899 |
|  | **FCFP6** | **0.918** |

**Figure S2.** Scaffolds predicted bioactive by the SVM and/or Bayesian classification models that are not present in the training set but have documented activity against *Plasmodium spp*. Their biological targets have not yet been elucidated.

**Table S5.** Summarised in silico and experimental results for 31 compounds purchased from ChemDiv.

| **Compound code** | **Structure** | **Vina binding affinity**  **(kcal mol^-1^)** | **Antiparasite activity prediction score** | **NP-40 βH IC_50_ (µM)** | **% *Pf* inhibition** |
| --- | --- | --- | --- | --- | --- |
| **O1** |  | -12.1 | 0.60^a^  8.98^b^ | >500 | <50^c^  <50^d^ |
| **O2** |  | -12.9 | 0.69^a^  5.70^b^ | >500 | <50^c^  <50^d^ |
| **O3** |  | -12.3 | 0.92^a^  9.45^b^ | 8 | 100^c^  100^d^ |
| **O4** |  | -12.9 | 0.62^a^  13.9^b^ | >500 | <50^c^  <50^d^ |
| **O5** |  | -12.4 | 0.92^a^  10.2^b^ | 129 | 68^c^  <50^d^ |
| **O6** |  | -13.4 | 0.85^a^  6.17^b^ | >500 | <50^c^  <50^d^ |
| **O7** |  | -12.2 | 0.61^a^  21.3^b^ | >500 | 78^c^  <50^d^ |
| **O8** |  | -12.0 | 0.89^a^  7.78^b^ | 129 | 100^c^  50^d^ |
| **B1** |  | -13.5 | 0.43^a^  8.34^b^ | 37 | 100^c^  <50^d^ |
| **B2** |  | -14.1 | 0.21^a^  6.14^b^ | 24 | 100^c^  <50^d^ |
| **B3** |  | -15.8 | 0.07^a^  4.86^b^ | >500 | 61^c^  <50^d^ |
| **B4** |  | -12.1 | 0.17^a^  6.87^b^ | >500 | 100^c^  <50^d^ |
| **B5** |  | -12.6 | 0.13^a^  10.5^b^ | >500 | <50^c^  <50^d^ |
| **B6** |  | -12.4 | 0.23^a^  8.67^b^ | >500 | 100^c^  <50^d^ |
| **B7** |  | -12.7 | 0.06^a^  5.53^b^ | >500 | <50^c^  <50^d^ |
| **B8** |  | -13.4 | 0.24^a^  4.90^b^ | >500 | <50^c^  <50^d^ |
| **S1** |  | -13.3 | 0.73^a^  -10.2^b^ | 30 | 100^c^  100^d^ |
| **S2** |  | -13.0 | 0.70^a^  2.73^b^ | 40 | <50^c^  <50^d^ |
| **S3** |  | -12.1 | 0.55^a^  1.59^b^ | 82 | 100^c^  <50^d^ |
| **S4** |  | -12.6 | 0.86^a^  1.50^b^ | 16 | 62^c^  <50^d^ |
| **S5** |  | -12.1 | 0.63^a^  -0.51^b^ | >500 | <50^c^  <50^d^ |
| **S6** |  | -12.7 | 0.58^a^  1.89^b^ | >500 | <50^c^  <50^d^ |
| **S7** |  | -13.1 | 0.82^a^  2.02^b^ | >500 | <50^c^  <50^d^ |
| **I1** |  | -12.5 | 0.00  -10.6 | >500 | <50^c^  <50^d^ |
| **I2** |  | -4.9 | nd | >500 | <50^c^  <50^d^ |
| **I3** |  | -12.2 | 0.02  -33.1 | 15 | <50^c^  <50^d^ |
| **I4** |  | -5.5 | nd | >500 | <50^c^  <50^d^ |
| **I5** |  | -4.5 | nd | >500 | <50^c^  <50^d^ |
| **I6** |  | -12.1 | 0.01  -30.1 | 11 | <50^c^  <50^d^ |
| **I7** |  | -4.7 | nd | >500 | <50^c^  <50^d^ |
| **I8** |  | -12.3 | 0.00  -11.6 | >500 | 57  59 |
| **chloroquine** |  | -8.5 | 0.06  9.2 | 25 | 2.9  0 |

Antiparasite prediction score: ^a^ SVM probability

^b^ Bayesian score

% *Pf* inhibition at: ^c^ 5 µM

^d^ 1 µM

**Table S6.** NP-40 βH formation assay results for 31 compounds purchased from ChemDiv.

| **Plate** | **Cpd ID** | **IC_50_ (µM)** | | | | **Std Dev**  **(µM)** | **Std Error (SEM, (µM))** |
| --- | --- | --- | --- | --- | --- | --- | --- |
|  |  | **Rep 1** | **Rep 2** | **Rep 3** | **Average** |  |  |
| 1 | **O1** | >500 | | | 500 |  |  |
|  | **O3** | 8.0 | 6.7 | 8.1 | 8 | 1 | 0.4 |
|  | **O5** | 128.6 | 125.9 | 133.0 | 129 | 4 | 2.1 |
|  | **CQ** | 32.7 | 25.8 | 31.8 | 30 | 4 | 2.2 |
| 2 | **O7** | >500 | | | 500 |  | 0.0 |
|  | **O8** | 133.0 | 128.3 | 127.0 | 129 | 3 | 1.8 |
|  | **B1** | 38.3 | 37.4 | 33.9 | 37 | 2 | 1.3 |
|  | **CQ** | 27.5 | 29.3 | 31.0 | 29 | 2 | 1.0 |
| 3 | **B2** | 25.4 | 21.1 | 24.9 | 24 | 2 | 1.4 |
|  | **B5** | >500 | | | 500 |  |  |
|  | **B6** | >500 | >500 | 172.2 | 500 |  |  |
|  | **CQ** | 23.9 | 30.1 | 22.7 | 26 | 4 | 2.3 |
| 4 | **B7** | >500 | | | 500 |  | 0.0 |
|  | **S1** | 31.9 | 32.8 | 24.2 | 30 | 5 | 2.7 |
|  | **S2** | 34.6 | 43.0 | 42.5 | 40 | 5 | 3.3 |
|  | **CQ** | 21.4 |  | 26.1 | 24 | 3 | 2.4 |
| 5 | **S3** | 82.6 | 96.8 | 65.6 | 82 | 16 | 11.0 |
|  | **S4** | 13.6 | 16.3 | 16.9 | 16 | 2 | 1.3 |
|  | **S5** | >500 | | | 500 |  |  |
|  | **CQ** | 14.4 | 14.6 | 14.1 | 14 | 0 | 0.2 |
| 6 | **S6** | >500 | | | 500 |  |  |
|  | **S7** | >500 | | | 500 |  |  |
|  | **I1** | >500 | | | 500 |  |  |
| 7 | **I3** | 15.6 | 15.4 | 13.0 | 15 | 1 | 0.8 |
|  | **I6** | 9.0 | 14.1 | 9.6 | 11 | 3 | 1.6 |
|  | **I8** | >500 | >500 | >500 | 500 |  |  |
|  | **CQ** | 25.6 | 35.1 | 30.7 | 30 | 5 | 2.7 |

**Table S7.** Single-point pLDH results for 31 compounds purchased from ChemDiv against the NF54 strain of *Plasmodium falciparum.* Compounds which inhibit parasite survival by greater than 50% are highlighted in green/orange.

| **% Parasite survival at 1 μM** | | | | **% Parasite survival at 5 μM** | | | |
| --- | --- | --- | --- | --- | --- | --- | --- |
|  |  |  | Average % |  |  |  | Average % |
| **O1** | 82.0 | 106.9 | 94.4 | **O1** | 74.6 | 68.6 | 71.6 |
| **O2** | 102.8 | 114.9 | 108.8 | **O2** | 39.2 | 66.0 | 52.6 |
| **O3** | -1.8 | -7.8 | -4.8 | **O3** | -13.5 | -14.3 | -13.9 |
| **O4** | 104.0 | 81.2 | 92.6 | **O4** | 82.5 | 78.2 | 80.3 |
| **O5** | 93.7 | 91.5 | 92.6 | **O5** | 55.2 | 9.5 | 32.3 |
| **O6** | 101.8 | 115.9 | 108.8 | **O6** | 59.9 | 64.6 | 62.2 |
| **O7** | 122.1 | 98.2 | 110.2 | **O7** | 17.8 | 26.8 | 22.3 |
| **O8** | 35.5 | 66.1 | 50.8 | **O8** | -0.2 | -6.9 | -3.5 |
|  |  |  |  |  |  |  |  |
| **B1** | 39.1 | 82.5 | 60.8 | **B1** | -1.4 | 0.8 | -0.3 |
| **B2** | 49.7 | 103.9 | 76.8 | **B2** | -5.2 | -9.5 | -7.3 |
| **B3** | 95.9 | 122.0 | 109.0 | **B3** | 13.6 | 64.2 | 38.9 |
| **B4** | 95.9 | 106.1 | 101.0 | **B4** | -6.5 | -10.5 | -8.5 |
| **B5** | 89.1 | 103.7 | 96.4 | **B5** | 70.4 | 59.7 | 65.1 |
| **B6** | 115.6 | 104.2 | 109.9 | **B6** | -8.9 | -13.9 | -11.4 |
| **B7** | 98.5 | 115.2 | 106.9 | **B7** | 54.0 | 60.9 | 57.5 |
| **B8** | 92.6 | 65.8 | 79.2 | **B8** | 72.3 | 64.6 | 68.5 |
|  |  |  |  |  |  |  |  |
| **S1** | -9.9 | 5.8 | -2.0 | **S2** | -15.5 | -30.1 | -22.8 |
| **S2** | 98.5 | 79.4 | 88.9 | **S3** | 73.7 | 68.7 | 71.2 |
| **S3** | 73.4 | 73.5 | 73.4 | **S4** | -3.4 | 2.7 | -0.4 |
| **S4** | 103.5 | 94.1 | 98.8 | **S5** | 35.3 | 39.7 | 37.5 |
| **S5** | 100.6 | 95.4 | 98.0 | **S6** | 74.9 | 84.7 | 79.8 |
| **S6** | 51.5 | 109.1 | 80.3 | **S7** | 82.5 | 78.7 | 80.6 |
| **S7** | 75.3 | 36.0 | 55.7 | **S8** | 62.4 | 81.1 | 71.7 |
|  |  |  |  |  |  |  |  |
| **I1** | 16.3 | 99.6 | 58.0 | **I1** | 63.2 | 94.5 | 78.9 |
| **I2** | 81.7 | 114.4 | 98.1 | **I2** | 55.2 | 46.8 | 51.0 |
| **I3** | 75.5 | 86.3 | 80.9 | **I3** | 81.9 | 38.3 | 60.1 |
| **I4** | 84.0 | 97.5 | 90.7 | **I4** | 86.1 | 60.6 | 73.3 |
| **I5** | 79.6 | 57.7 | 68.7 | **I5** | 65.6 | 75.7 | 70.6 |
| **I6** | 104.2 | 118.3 | 111.2 | **I6** | 86.4 | 69.8 | 78.1 |
| **I7** | 21.9 | 65.1 | 43.5 | **I7** | 89.2 | 80.5 | 84.8 |
| **I8** | 50.9 | 30.8 | 40.9 | **I8** | 31.9 | 53.8 | 42.8 |
|  |  |  |  |  |  |  |  |
| **CQ** |  |  | 0.0 | **CQ** |  |  | 2.9 |

**Table S8.** In vitro antiplasmodium activity of selected compounds tested against the CQ-sensitive (NF54) and resistant (Dd2) strains of *P. falciparum.*

| **Compound ID** | **IC_50_ ± SD (μM) – NF54^a^** | **IC_50_ ± SD (μM) – Dd2^a^** | **RI^b^** |
| --- | --- | --- | --- |
| **O3** | 0.154 ± 0.03 | 0.244 ± 0.002 | 1.6 |
| **O8** | 3.545 ± 0.26 | 3.320 ± 0.05 | 0.9 |
| **B1** | 1.610 ± 0.16 | 0.971 ± 0.018 | 0.6 |
| **B2** | 2.171 ± 0.63 | 3.010 ± 0.35 | 1.4 |
| **S1** | 0.158 ± 0.02 | 0.545 ± 0.05 | 3.4 |
| **S3** | 2.08 ± 0.14 | 1.78 ± 0.37 | 0.86 |
| **S4** | 6.14 ± 0.48 | 5.8 ± 1.3 | 094 |
| **CQ** | 0.014 ± 0.003 | 0.376 ± 0.03 | 26.7 |

^a^ Average IC50 values ± standard deviation (N=2, n=2)

^b^ Resistance Index = IC_50_(Dd2)/IC_50_(NF54)

**Experimental Methods**

**Detergent-mediated NP-40 assay for β-haematin (βH) formation**

The βH inhibition activity of the purchased compounds was investigated using the detergent-mediated NP-40 assay, developed by Carter et al. Stock solutions (10 mM) of the test compounds and a chloroquine diphosphate control were prepared in DMSO and water, respectively. Water (140 µL) and NP-40 detergent (305.5 µM, 40 µL) were added to column 12 of a 96-well plate. A 7:2:1 (v/v) solution of water/ NP-40 (305.5 µM)/ DMSO (100 µL) was added to columns 1-11. The test and control compounds (20 µL) were added in duplicate to column 12 and serially diluted through to column 2, leaving column 1 as a blank. A stock solution of haematin (25 mM) was prepared in DMSO and sonicated for 1 min. The haem stock (178.8 µL) was suspended in 2 M acetate buffer (20 mL, pH 4.8) and the suspension (100 µL) was added to each well to give final buffer and haematin concentrations of 0.5 M and 100 µM, respectively. The plate was incubated for 5 h at 37 °C. The assay was analysed using the pyridine-ferrochrome method, developed by Ncokazi and Egan. A 5:2:2:1 (v/v) solution of pyridine/water/acetone/HEPES buffer (2 M, pH7.4) was added (32 µL) to each well to give a final pyridine concentration of 5% (v/v). Acetone (60 µL) was added to assist haematin dispersion. The UV-vis absorbance of the plate was read at 405 nm on a Thermo Scientific Multiskan GO plate reader. The IC_50_ of each compound was calculated by plotting sigmoidal dose-response curves in GraphPad Prism v 9.0.0. (GraphPad Software Inc., La Jolla, CA, USA).

**Parasite lactate dehydrogenase (pLDH) assay for antiplasmodium activity**

Various strains of *P. falciparum* were maintained in continuous culture *in vitro* at 2% haematocrit and 5-10% parasitaemia following the methods of Trager and Jensen. The antimalarial activity of all test compounds was determined using a modified version of the parasite lactate dehydrogenase (pLDH) assay as described by Makler *et al*. All compounds were screened against both the chloroquine-sensitive NF54 and chloroquine-resistant Dd2 strains of *P. falciparum* with the exception of the single-point screen which was performed using the NF54 strain only. Stock solutions of test compounds were prepared at 10 mM in 100% DMSO. Chloroquine diphosphate was used as a control on all plates and was prepared at 20 mM in MilliQ water. All stock solutions were stored at -20°C. Compounds were diluted in culture medium to working concentrations of 40, 20, 10 or 2 μM respectively and then diluted two-fold using culture medium in 96-well plates. In the single-point screen, all samples were screened at 1 and 5 μM and a full dose-response was then performed on a selected set of compounds. To obtain IC50 values, all samples were tested in duplicate at ten different concentrations. The blank column contained unparasitised red blood cells at 1% haematocrit. The column representing the positive control (containing no test compound) and all wells inoculated with various compounds contained parasitised red blood cells at 1% haematocrit and 2% parasitaemia. The plates were incubated at 37°C for 48 h in a gas chamber comprising 3% O2, 4% CO2 and 93% N2. Following incubation, the plates were frozen at -20°C overnight and then thawed after which cell material in all wells were resuspended. The plates were developed by transferring 20 μL of cell material into corresponding wells of a 96-well plate containing 100 μL MALSTAT. Thereafter, 25 μL of NBT was added to each well and the absorbance was measured at 620 nm using the MultiSkan GO plate reader. The IC50 value for each compound was determined using the non-linear dose-response curve fit in GraphPad Prism v 9.0.0.
